# Parliament and the Professions

**Published:** 1921-06-18

**Authors:** 


### Parliament and the Professions.

### Beds in Ruskin Park.

The Minister of Pensions was asked a question as regards the use of accommodation for discharged Service men in Ruskin Park, and whether the patients could not be sent to vacant wards in hospitals. Mr. Macpherson stated that it was largely a matter of equipment, as the huts in question had been fitted with special orthopædic fittings, and there was a special section for discharged officers and nurses.

### The London Hospital.

A question was addressed to the Minister of Health as to the closing of wards at the London Hospital, and it was asked whether it was proposed to take any steps to provide adequate facilities for medical treatment for the people of East London. The reply was that the Government had not had an opportunity of considering Lord Cave's report, but there was accommodation for emergency cases in the Whitechapel and other infirmaries in East London.

### Training of Nurses and Midwives.

The Minister of Labour was asked whether any of the women under training as nurses had been rejected after trial as being unsuited for the nursing profession, and whether, in any of these cases, the Ministry had refused to accept this decision and continued the grants. Dr. Macnamara replied that no women had been placed in training as nurses by the Ministry of Labour, though a number are being trained as midwives. It is the practice to discontinue training immediately if the trainee is reported by the authorities to be unsuitable.

